# Establishment of an Efficient *Agrobacterium rhizogenes*-Mediated Hairy Root Transformation System for Functional Analysis in Passion Fruit

**DOI:** 10.3390/plants14152312

**Published:** 2025-07-26

**Authors:** Jiayi Pan, Yiping Zheng, Tiancai Wang, Pengpeng Xiong, Kaibo Cui, Lihui Zeng, Ting Fang

**Affiliations:** 1 College of Horticulture, Institute of Genetics and Breeding in Horticultural Plants, Fujian Agriculture and Forestry University, Fuzhou 350002, China; pan1161621228@163.com (J.P.); yiping0108@126.com (Y.Z.); 16635025885@163.com (T.W.); littlexiongzzz@163.com (P.X.); cuikaibo114514@163.com (K.C.); 2 Institute of Biotechnology, Fujian Academy of Agricultural Sciences, Fuzhou 350003, China; 3 Key Laboratory of Ministry of Education for Genetics, Breeding and Multiple Utilization of Crops, Fujian Agriculture and Forestry University, Fuzhou 350002, China

**Keywords:** passion fruit, *Agrobacterium rhizogenes*, hairy root, *PeMYB123*, proanthocyanidin

## Abstract

Passion fruit (*Passiflora edulis* Sims), belonging to the Passifloraceae family, is an economically important plant in tropical and subtropical regions. The advances in functional genomics research of passion fruit have been significantly hindered by its recalcitrance to regeneration and stable transformation. This study establishes the first efficient *Agrobacterium rhizogenes*-mediated hairy root transformation system for passion fruit. Utilizing the *eGFP* marker gene, transformation efficiencies of 11.3% were initially achieved with strains K599, MSU440, and C58C1, with K599 proving most effective. Key transformation parameters were systematically optimized to achieve the following: OD_600_ = 0.6, infection duration 30 min, acetosyringone concentration 100 μM, and a dark co-cultivation period of 2 days. The system’s utility was further enhanced by incorporating the red visual marker RUBY, enabling direct, instrument-free identification of transgenic roots via betaxanthin accumulation. Finally, this system was applied for functional analysis using *PeMYB123*, which may be involved in proanthocyanidin accumulation. Overexpression of *PeMYB123* produced a higher content of proanthocyanidin in hairy roots. Additionally, the *PeANR* gene involved in the proanthocyanidin pathway was strongly activated in the transgenic hairy roots. This rapid and efficient visually simplified hairy root transformation system provides a powerful tool for functional gene studies in passion fruit.

## 1. Introduction

Passion fruit (*Passiflora edulis* Sims), also known as egg fruit, lover’s fruit, purple passion fruit, or native passion fruit, belongs to the Passifloraceae family and *Passiflora* genus [1]. Native to the Greater and Lesser Antilles, it is now widely cultivated in tropical and subtropical regions worldwide [2]. Due to its significant role in rural revitalization, passion fruit cultivation has expanded rapidly in China in recent years, primarily in Guangxi, Fujian, Yunnan, and Hainan provinces [3]. Passion fruit possesses notable edible, medicinal, and ornamental value. Nutritionally, it is rich in numerous compounds, including proteins, sugars, vitamins, potassium, and amino acids, earning it titles like “King of Fruits” and “King of Vitamin C” [4]. Medicinally, extracts from its leaves, fruits, rind, and seeds exhibit antioxidant, anti-inflammatory, anti-cancer, anti-diabetic, anti-hypertensive, and anti-aging properties [5]. Ornamentally, the plant features large, fragrant flowers with bright coronal filaments and intricate nectariferous structures, complemented by luxuriant foliage [6]. Despite significant advances in passion fruit molecular research enabled by genomic tools over the past decade, functional gene characterization remains impeded by the lack of an efficient genetic transformation system.

Proanthocyanidin (PA), or condensed tannin, belongs to a class of polyphenolic compounds widely distributed in the plant kingdom [7]. Abundant in plant skins, seeds, barks, and leaves, major PA sources include grape (*Vitis vinifera*) seeds, pine bark (*Pinus pinaster*), blueberries (*Vaccinium* spp.), and cocoa beans [8]. Although passion fruit peel exhibits lower PA levels than blueberries, these secondary metabolites demonstrate significant bioaccumulation in the pericarp layer, establishing them as functionally important phytochemicals in this tropical species [9]. PAs are notably potent antioxidants, scavenging free-radicals 20–50 times more effectively than vitamins C and E [10]. Their key functional roles in human health encompass cardioprotective properties, anti-inflammatory and anti-cancer effects, diabetes management, dermatological benefits, and neuroprotection [11].

The PAs biosynthesis proceeds via the phenylpropanoid pathway, initiating from phenylalanine and involving key enzymes such as chalcone synthase (CHS), flavanone-3-hydroxylase (F3H), anthocyanidin reductase (ANR), and leucoanthocyanidin reductase (LAR) [12]. This process is further regulated at the transcriptional level by transcription factors (TFs), particularly the MYB-bHLH-WD40 (MBW) ternary complex, which modulate structural gene expression [13]. Within this complex, MYB transcription factors play a pivotal regulatory role. In apple callus, overexpression of *MdMYB9* and *MdMYB11* significantly enhanced PA accumulation while markedly upregulating *ANR* and *LAR* expression [14]. In grape, VvMYBPA1 and VvMYBPA2 simultaneously activate *LAR* and *ANR* genes, promoting PA biosynthesis [15]. Conversely, in peach, PpMYBPA1 and PpMYB7 specifically regulate PA synthesis by activating *PpLAR1* expression but show no significant effect on *PpANR* [16].

To enhance passion fruit production quality and crop value, genomic intervention is imperative, with the establishment of an efficient genetic transformation protocol serving as its cornerstone [17]. Since the creation of the first genetically modified organism (GMO) in 1983, plant transgenic technology has advanced significantly. Various plant transformation methods have been developed, including electroporation, gene gun bombardment, pollen tube pathway, and *Agrobacterium*-mediated transformation [18]. Among these, *Agrobacterium*-mediated transformation is the most widely adopted, accounting for approximately 85% of existing transgenic plants [19]. Two primary *Agrobacterium* species are utilized: *Agrobacterium tumefaciens* and *A. rhizogenes* [20]. *A. tumefaciens*-mediated transformation is particularly prevalent in species like apple [21], citrus [22], and grape [23] due to its operational simplicity, cost-effectiveness, and tendency for low-copy-number transgene integration. However, this method faces limitations including low transformation efficiency, variable recipient cell sensitivity, recalcitrant regeneration, and unpredictable transgene integration sites. In passion fruit, initial transgenic plants were obtained using leaf and stem explants [24]. Recent progress has leveraged transient *Agrobacterium* transformation to study gene function, such as *PeCWINV5*’s role in sucrose unloading and hexose accumulation [25]. Transgenic lines expressing viral coat protein genes (e.g., *CABMV-CP*, PMV-CP) have shown reduced susceptibility to pathogens like cowpea aphid-borne mosaic virus (CABMV) and passion fruit woodiness virus (PMV), demonstrating the potential for engineered disease resistance [26,27]. Despite these advances, a robust, efficient stable transformation system for passion fruit based on *A. tumefaciens* remains elusive. The development of alternative transformation strategies is therefore critically needed.

*A. rhizogenes* (*Rhizobium rhizogenes*), a Gram-negative bacterium with a broad host range, carries T-DNA on its Ri plasmid. In 1974, scientists first discovered that the Ri plasmid carried by *A. rhizogenes* can induce plant cells to produce abnormally proliferating root-like structures, namely hairy roots [28]. In the 1980s, due to their advantages such as rapid growth, high genetic stability, and efficient synthesis of secondary metabolites, hairy roots were gradually applied to the research of medicinal plants [29]. In the 1990s, by means of *A. rhizogenes*-mediated transformation technology, target genes were introduced into hairy roots. Additionally, the hairy root culture system began to be used in the research on the interaction mechanism between plants and microorganisms [30]. Hairy roots offer advantages in genome research such as efficient genetic transformation, good genetic stability, rapid growth with simple culture conditions, convenience for genome editing operations, and the ability to reflect the genomic characteristics of plant roots [28,30]. Nowadays, *A. rhizogenes*-mediated transformation technology has been established in diverse horticultural species, such as apple (*Malus domestica*) [31], woodland strawberry (*Fragaria vesca*) [32], and litchi (*Litchi chinensis*) [33]. Notably, hairy root induction efficiency varies substantially (2–88.3% in woody plants), highlighting methodological influences on transformation success.

In this study, we report the first development of a rapid and highly efficient *A. rhizogenes*-mediated hairy root transformation system for passion fruit. We systematically evaluated and optimized key transformation parameters, including *A. rhizogenes* strain selection, bacterial suspension concentration, infection duration, acetosyringone (AS) concentration, and dark culture period, using the enhanced green fluorescent protein (eGFP) reporter. Furthermore, the RUBY visual marker system was employed to enable the equipment-free identification of transgenic hairy roots. To demonstrate the system’s utility for functional genomics, we successfully characterized *PeMYB123*—a key transcription factor regulating proanthocyanidin biosynthesis. This optimized transformation platform provides an effective and streamlined methodology for passion fruit gene functional studies and establishes a foundation for broader applications of *A. rhizogenes* technology in this crop.

## 2. Results

### 2.1. Hairy Root Induction of Passion Fruit Seedlings by A. rhizogenes

**Figure 1:**
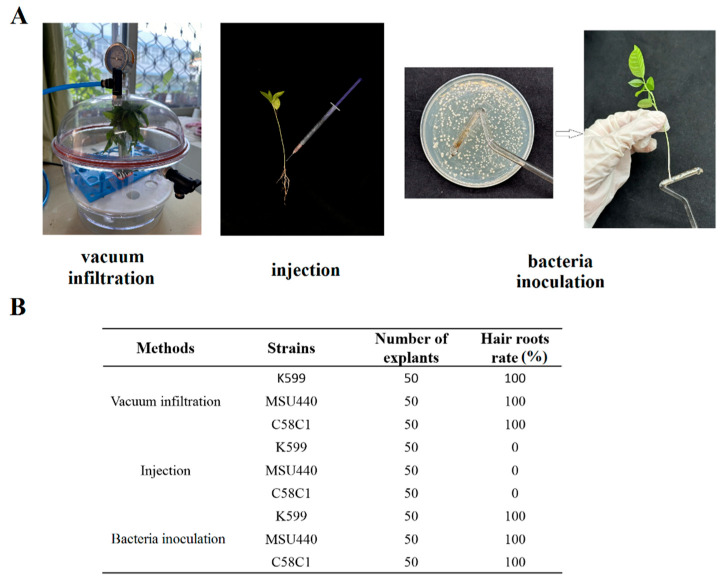
Effects of different infection methods on hairy roots induction rate in passion fruit. (**A**) Three infection methods. (**B**) Comparative analysis of the hairy roots induction rate of three infection methods.

In *A. rhizogenes*-mediated transformation, infection site and methodology critically impact induction efficiency. Four-week-old seedlings were subjected to the following three transformation methods: (1) vacuum infiltration, (2) bacterial inoculation, and (3) needle injection (Figure 1A). The hairy root can be observed within approximately 7–10 days. After one month, both vacuum infiltration and bacterial inoculation methods achieved 100% hairy root induction at root collars (Figure 1B). In terms of morphology, hairy roots have unique characteristics; they usually appear as dense, slender, and highly branched structures with vigorous growth, while the roots of untransformed plants typically have a distinct main root, with lateral roots growing orderly from the main root, showing an overall regular structure with relatively few branches and far fewer root hairs than hairy roots (Figure S1). Conversely, needle injection caused significant tissue damage, resulting in wound necrosis that progressed to plant wilting and death. Following the removal of necrotic specimens, this method yielded only 1% induction efficiency (Figure 1B). While induction rates were equivalent for vacuum infiltration and bacterial inoculation, vacuum infiltration demonstrated superior operational efficiency with reduced handling requirements. This method was consequently selected for subsequent transformations.

### 2.2. A. rhizogenes Strain K599 Was the Most Suitable Strain for Hairy Root Transformation in Passion Fruit

**Figure 2:**
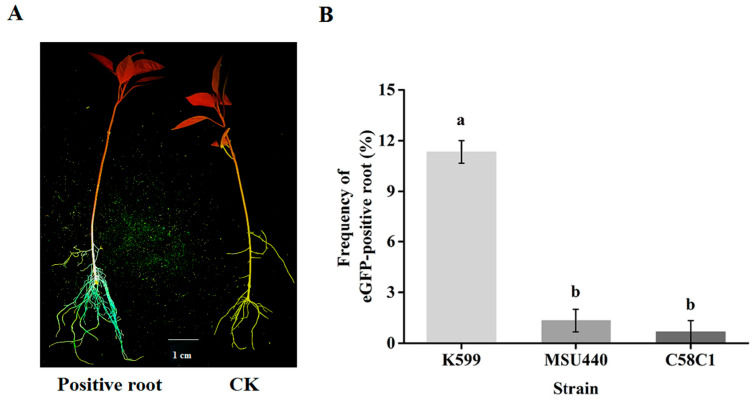
Effects of different types of *A. rhizogenes* strains on the hairy root transformation of passion fruit. (**A**) Transgenic roots identification based on eGFP (enhanced green fluorescent protein). (**B**) Comparative analysis of the genetic transformation efficiency of three *A. rhizogenes* strains. The visualization of eGFP fluorescence in transgenic hairy roots was detected using a LUYOR-3415RG dual florescent protein flashlight (excitation wavelength 440 nm and emissions wavelength 500 nm). The values are presented as the mean ± SE. Different alphabetical letters indicate statistical significance at *p* < 0.05 (1-way ANOVA with Tukey’s test).

To identify optimal strains for passion fruit hairy root transformation, we evaluated three widely used *A. rhizogenes* strains: K599, MSU440, and C58C1. Each strain carried the binary vector pH7LIC5.0-ccdB rc-NeGFP expressing eGFP under the CaMV 35S promoter. Under standardized transformation conditions (vacuum infiltration method), K599 demonstrated superior performance, with intense GFP fluorescence observed throughout transgenic roots (Figure 2A). Quantitative analysis revealed significantly higher transformation efficiency (11.3% eGFP-positive roots) for K599 compared to MSU440 (2%) and C58C1 (1%) (Figure 2B). Consequently, K599 was selected for all subsequent passion fruit transformations.

### 2.3. PCR Verification of eGFP-Positive Roots

**Figure 3:**
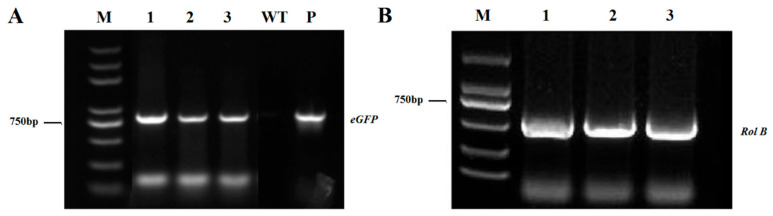
PCR verification of *A. rhizogenes*-mediated hairy root transformation of passion fruit. (**A**) PCR analysis for *eGFP* in three independent transgenic roots. M, 5 kb DNA ladder marker; P: 35S::eGFP plasmid DNA; WT, non-transgenic DNA (negative control); 1–3, three independent transgenic hairy roots. (**B**) PCR analysis for *Rol B* in three independent transgenic roots. M, 2 kb DNA ladder marker; 1–3, three independent transgenic hairy roots.

PCR analysis was also performed on randomly selected independent plants to confirm the transgenic status of the hairy roots. The 35S::eGFP plasmid served as the positive control, while non-transgenic roots were used as the negative control. Bands corresponding to *rol B* and *eGFP* were absent in the negative control. However, these bands were clearly detected in the transgenic hairy roots exhibiting eGFP fluorescence, demonstrating successful integration of the plasmid fragment into the genome of the transgenic hairy roots (Figure 3). In summary, the *A. rhizogenes*-mediated hairy root transformation system proved amenable for passion fruit.

### 2.4. Optimization of the Hairy Root Transformation System in Passion Fruit

**Figure 4:**
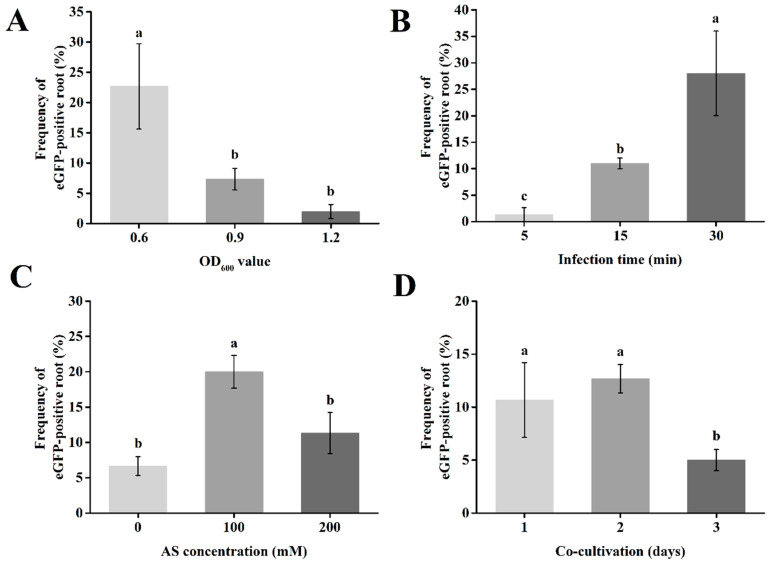
Optimizing the *A. rhizogenes*-mediated hairy root transformation of passion fruit. The eGFP-positive rates were compared after infection with *A. rhizogenes* strain K599 at different OD_600_ values (**A**), infection times (**B**), AS concentration (**C**), and co-cultivation times (**D**), respectively. Different letters indicate significant differences (*p* ≤ 0.05).

To optimize *A. rhizogenes* co-transformation efficiency, the following key parameters were tested: bacterial concentration (OD_600_), infection time, acetosyringone (AS) concentration, and dark culture duration. One-month seedlings were infected with the *A. rhizogenes* strain K599, and the eGFP-positive root rate was evaluated. First, a gradient of *A. rhizogenes* concentrations (OD_600_ = 0.6–1.2) was compared. Transformation efficiency decreased significantly with increasing bacterial concentration (Figure 4A). The highest frequency of eGFP-positive hairy roots (22.67%) occurred at OD_600_ = 0.6, suggesting this as the optimal concentration for passion fruit transformation. Subsequently, infection times (5–30 min) were tested at OD_600_ = 0.6. Transformation efficiency increased significantly from 5 to 30 min, peaking at 30 min (Figure 4B).

Additionally, different acetosyringone (AS) concentrations (0, 100, and 200 μM) were tested. Seedlings were infected with *A. rhizogenes* at OD_600_ = 0.6 for 30 min prior to AS application. As shown in Figure 4C, the frequency of eGFP-positive roots increased significantly from 0 to 100 μM AS, but decreased significantly at 200 μM. The highest transformation frequency (20%) was observed at 100 μM AS. Finally, different co-cultivation durations (1, 2, and 3 days) were tested. Seedlings were infected under the following optimized conditions: *A. rhizogenes* at OD_600_ = 0.6, infection time of 30 min, and 100 μM AS. The results showed that the frequency of eGFP-positive roots was significantly higher after 2 days of co-cultivation compared to 1 or 3 days (Figure 4D). Collectively, these results demonstrate that the optimized transformation conditions are as follows: *A. rhizogenes* concentration at OD_600_ = 0.6, infection time of 30 min, AS concentration of 100 μM, and co-cultivation duration of 2 days.

### 2.5. Gene Functional Analysis of PeMYB123 Using the Hairy Root Transgenic System

**Figure 5:**
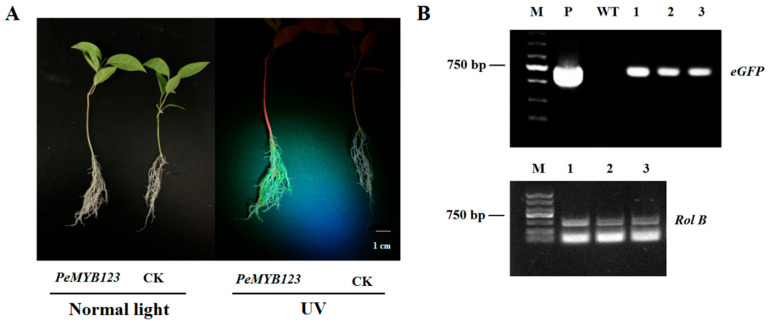
Identification of transgenic hairy roots overexpressing *PeMYB123* in passion fruit. (**A**) Transgenic roots identification based on eGFP. (**B**) PCR analysis of *eGFP* and *rol B* in independent transgenic hairy roots for validation of overexpressing *PeMYB123*.M, 2 kb DNA ladder marker; P, 35S::eGFP plasmid DNA; WT, non-transgenic DNA (negative control); 1-3, three independent transgenic hairy roots.

Based on transcriptome data from different developmental stages of purple passion fruit [34], we identified elevated expression of P_edulia020006262.g at the mature stage compared to the green stage. Phylogenetic analysis indicated that P_edulia020006262.g shares the highest homology with AtMYB123 (Figure S2) and was consequently designated PeMYB123. Further phylogenetic analysis revealed that PeMYB123 clusters within the PA activator group (Figure S3), suggesting its potential role as a transcriptional activator of PA biosynthesis in passion fruit. To investigate the function of *PeMYB123* in PA accumulation, the *PeMYB123*-eGFP construct was introduced into the *A. rhizogenes* strain K599 and used for hairy root transformation. Strong GFP fluorescence throughout the transgenic hairy roots was observed 30 days post-transformation (Figure 5A). To confirm the transgenic status, PCR analysis was performed on three randomly selected, independent transgenic hairy root lines. Sequences encoding eGFP and the rolB protein were detected in all lines (Figure 5B), confirming successful integration of the *PeMYB123*-eGFP vector into the passion fruit hairy root genome.

**Figure 6:**
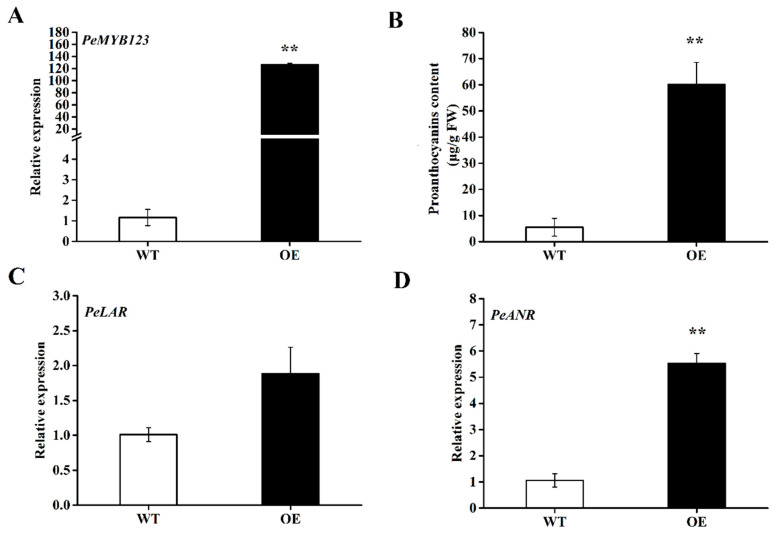
Functional analysis of *PeMYB123* in passion fruit hairy roots. (**A**) Relative expression of *PeMYB123* in wild-type (WT) and transgenic hairy roots (OE). (**B**) Measurement of proanthocyanidin content in WT and OE hairy roots. (**C**) Relative expression of *PeLAR* in WT and OE hairy roots. (**D**) Relative expression of *PeANR* in WT and OE hairy roots. Asterisks indicate significant differences (** *p* ≤ 0.01).

Furthermore, qRT-PCR analysis revealed significantly higher transcript abundance of *PeMYB123* in the OE-PeMYB123 hairy roots compared to the control (Figure 6A). Quantification of PA content showed an 11-fold increase in the OE-*PeMYB123* lines relative to the control (Figure 6B). To elucidate the regulatory role of *PeMYB123*, we analyzed the expression of key flavonoid pathway target genes, *PeLAR* and *PeANR*, using qRT-PCR. The results demonstrated specific upregulation of *PeANR* expression, while *PeLAR* remained significantly unchanged (Figure 6C,D). These findings demonstrate that *PeMYB123* functions as a transcriptional activator, promoting PA accumulation through the specific induction of PA biosynthetic genes (*PeANR*) in passion fruit. Collectively, this study establishes the *A. rhizogenes*-mediated hairy root system as an effective platform for studying PA metabolism and enables rapid functional validation of genes in passion fruit, a semi-woody species.

### 2.6. Application of RUBY in Hair Root Transformation System of Passion Fruit

**Figure 7:**
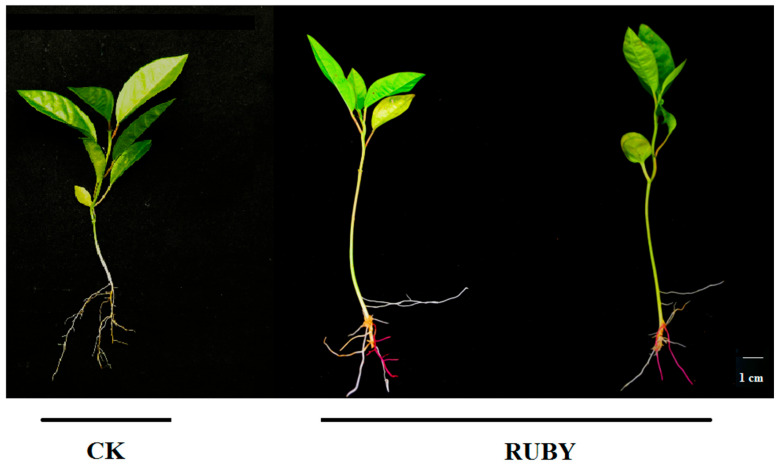
RUBY functioned as an effective and visible reporter in passion fruit.

Traditional chromogenic detection of marker genes often requires sophisticated instruments, costly reagents, or invasive chemical treatments. In contrast, the RUBY reporter system enables the non-invasive visualization of gene expression without chemical processing or specialized equipment. To validate this reporter, we constructed a plant expression vector harboring the *RUBY* gene and introduced it into *A. rhizogenes*. This engineered strain was then used to infect passion fruit explants, inducing transgenic hairy root formation. Under appropriate culture conditions, infected explants developed new roots. Within four weeks, a subset of these roots exhibited an intense red coloration (Figure 7), directly indicating successful *RUBY* gene expression. This vivid phenotype is readily visible to the naked eye, eliminating the need for handheld fluorometers or other detection devices. The RUBY system thus significantly streamlines the screening process for transgenic roots and markedly improves workflow efficiency.

## 3. Discussion

### 3.1. Establishment of A. rhizogenes-Mediated Hairy Root Transformation System in Passion Fruit

Over the past two decades, the rapid advancement of next-generation sequencing (NGS) technology has enabled the assembly of reference genomes for more than 30 fruit tree species. This genomic foundation paves the way for genetic engineering and molecular breeding aimed at crop improvement [35]. Consequently, identifying genes controlling key horticultural traits, such as yield, fruit quality, and stress resistance, has become increasingly feasible. In passion fruit, integrated transcriptomic and metabolomic analyses have identified several key genes involved in flavonoid biosynthesis [34,36]. However, the functional validation of these genes has largely been restricted to model plants like *Nicotiana benthamiana* [34,36,37] due to the absence of efficient transformation protocols for non-model species, significantly impeding functional genomics research in passion fruit. Therefore, establishing robust genetic transformation methods is essential to overcome this limitation. Although *A. rhizogenes*-mediated hairy root genetic transformation systems have been established in many fruit crops, no relevant reports have been found in passion fruit. The *A. rhizogenes*-mediated transgenic hairy root system developed in this study addresses this critical need and accelerates functional genomics in passion fruit.

Significant progress has recently been made in optimizing genetic transformation for fruit trees, including longan [38], grape [39], citrus [40], and kiwifruit [31]. While *Agrobacterium*-mediated transformation has been established in passion fruit, its low efficiency (0.11–5.7%) and prolonged timeframe [41] present substantial limitations for functional genomics. Although a recent study reported higher transformation efficiency (29%) using seedling stem cuttings [42], it relied solely on GUS staining, GFP visualization, and transgene expression analysis without phenotypic validation. Thus, *A. tumefaciens*-mediated transformation remains time-consuming, recalcitrant, and inefficient for high-throughput functional genomics. In contrast, *A. rhizogenes* has emerged as a valuable tool for functional gene studies, particularly those investigating secondary metabolism and abiotic stress responses [43,44]. Herein, we demonstrate an efficient, rapid, and stable *A. rhizogenes*-mediated transformation system for passion fruit using stem explants. This approach aligns with reports demonstrating the suitability of hypocotyl, leaf, and shoot explants for *A. rhizogenes* transformation in other species like peach.

### 3.2. Factors Influencing the A. rhizogenes-Mediated Hairy Root Transformation Efficiency in Passion Fruit

Multiple factors influence transformation efficiency, including *Agrobacterium* strain type, bacterial cell density (OD_600_), explant selection, AS concentration, infection time, and co-cultivation duration. *A. rhizogenes* strains are categorized into the following four types based on the opines synthesized by their Ri plasmids: mannopine, cucumopine, agropine, and mikimopine [45]. This study evaluated the following three strains: K599, C58C1, and MSU440. C58C1 and MSU440 harbor agropine-type Ri plasmids, while K599 contains a cucumopine-type Ri plasmid [45]. K599 demonstrated significantly higher transformation efficiency than C58C1 and MSU440 in passion fruit. This difference may be attributed to the Ri plasmid type, suggesting the cucumopine-type plasmid in K599 promotes hairy root transformation in this species. This observation aligns with findings in other woody plants like pigeon pea (*Cajanus cajan*) [46] and pecan (*Carya illinoinensis*) [47], where K599 also proved optimal. However, contrasting results were observed in woodland strawberry, where MSU440 and C58C1 outperformed K599 [32], indicating species-specific strain selectivity.

Bacterial density also critically impacts efficiency. Higher cell densities increase T-DNA transfer frequency by enhancing bacterial attachment to plant cells and subsequent T-DNA injection [48]. However, in our study using stem explants, transformation efficiency exhibited a steady decline with increasing OD_600_ values (0.6, 0.9, and 1.2). This contrasts with some reports as Cheng et al. [49] achieved 69% transformation in soybean using OD_600_ = 0.6 under tissue culture, while Yu et al. [50] reached 80% in *Brassica napus* using OD_600_ = 0.4–0.6 without tissue culture. Optimal concentrations vary significantly among woody species, exemplified by OD_600_ = 0.2–0.6 in pigeon pea [46] and OD_600_ = 0.8 in pecan [47].

Furthermore, AS enhances transformation efficiency by activating vir genes in *A. rhizogenes* [51], significantly improving hairy root induction when added to the bacterial suspension [52,53]. Our results confirmed this, showing significantly higher transformation efficiency with 100 μM AS compared to other concentrations. Despite these insights, numerous factors influencing the *Passiflora edulis* transformation system remain to be fully explored.

### 3.3. Application of the A. rhizogenes-Mediated Hairy Root Transformation Efficiency in Passion Fruit

Nowadays, the transgenic hairy roots system has been widely used in many aspects, such as stress response, metabolism, and root biology [33,54,55]. In grape, the system was successfully applied to investigate gene functions under low-temperature stress [39,44]. The production of genetically transformed hairy roots offers a promising alternative for producing target compounds in medicinal plants. For instance, transgenic *Salvia miltiorrhiza* hairy roots accumulated significantly higher levels of total tanshinones compared to field-grown plant roots [56,57]. In this study, a stable transformation system was established for the first time to validate the function of *PeMYB123* in regulating proanthocyanidin biosynthesis in passion fruit. Results demonstrated that overexpression of *PeMYB123* actively induced proanthocyanidin accumulation in hairy roots. Furthermore, qRT-PCR analysis revealed that *PeMYB123* upregulated the expression of *PeANR*, a key gene involved in the proanthocyanidin biosynthetic pathway. These findings confirm that the transgenic hairy root system serves as an effective platform for gene functional studies in passion fruit. Notably, this approach also enables the application of silencing techniques or gene editing—particularly for plants recalcitrant to in vitro regeneration [58,59]—warranting further exploration in passion fruit research.

Although this study successfully established a hairy root genetic transformation system for passion fruit and verified its effectiveness, there are certain limitations. Firstly, the system is currently established mainly based on seedling explants and obtained hairy roots, but regenerating complete plants from hairy roots is a challenging step, which has not been addressed. Secondly, although the functionality of the system was demonstrated through overexpression experiments of the *PeMYB123* gene, its compatibility with technologies such as gene silencing (e.g., RNAi) or editing (e.g., CRISPR/Cas9) has not been explored, which to some extent limits the comprehensive application of this system in reverse genetics research. In addition, the physiological status of hairy roots in this study may differ from that of whole plants, so the conclusions on gene function drawn from hairy roots need to be further verified in intact plants. Finally, although key transformation parameters were optimized, genetic background differences among different passion fruit varieties may affect the universality of the system. Future tests in more varieties are needed to improve the system.

## 4. Materials and Methods

### 4.1. Plant Materials and Growth Condition

After wrapping the seeds of passion fruit (Passiflora edulis Sims cv. ‘Fujian No. 3’) in gauze, rinse them under running water for 24 h. Then, sow the seeds in growth substrate (vermiculite:perlite:soil, 1:1:3 *v*/*v*/*v*) and maintain them at 25 ± 1 °C under a 16 h photoperiod.

### 4.2. Plasmids and Agrobacterium Bacterial Strains

The *A. rhizogenes* strains competent (K599, MSU440, and C58C1) were obtained from Koolab Biotechnology Co., Ltd. (Beijing, China). The red color vector RUBY plasmid, which includes the red visual marker gene, was obtained from Fenghui Biotechnology (Changsha, Hunan, China). The pH7LIC5.0-ccdB rc-N-eGFP vector, which includes the enhanced green fluorescent protein (eGFP) marker gene, was used for genetic transformation.

### 4.3. PeMYB123 Cloning

The coding sequence (CDS) of *PeMYB123* was cloned from the cDNA of purple passion fruit ‘Fujian No. 3’. The phylogenetic tree was constructed by the use of the neighbor-joining method in MEGA 7.0 software with a bootstrap value 1000.

The CDS of *PeMYB123* was inserted into the pH7LIC5.0-ccdB rc-N-eGFP vector (primers are listed in Table S1) and the recombinant plasmid was transformed into the *A. rhizogenes* K599 cells.

### 4.4. A. rhizogenes-Mediated Transformation

*A. rhizogenes* preparation: The pH7LIC5.0-ccdB rc-N-eGFP plasmid were transformed into *A. rhizogenes* strains (K599, MSU440, and C58C1) by the freeze–thaw method. Subsequently, 500 µL TY medium was added, and cultured at 28 °C for 2 h. The cells were then evenly spread on solid TY medium containing 50 mg·L^−1^ streptomycin and spectacular mycin and incubated at 28 °C for 2 days. Single colonies were picked and inoculated into 600 µL of liquid LB medium containing streptomycin and kanamycin, and they were cultured overnight at 28 °C with a shaking speed of 200 rpm. The bacterial solution was used in the PCR-based identification of transformed *Agrobacterium* lines. A total of 1 mL bacteria were transferred to 20 mL TY broth containing streptomycin and kanamycin (mg·L^−1^) and cultured at 28 °C, 200 rpm until OD_600_ = 0.6–1.2. The bacteria were collected by centrifugation at 6500 rpm at 4 °C for 10 min. Subsequently, the bacteria were suspended in infiltration solution (10 mM MES, 10 mM MgCl_2_, 100 μM AS, pH = 5.2) and the concentration was adjusted to OD_600_ = 0.6.

To induce hairy roots, the following three methods were used: vacuum infiltration, injection, and bacterial inoculation. For vacuum infiltration, the roots of the passion fruit seedlings were excised, and the explants were immersed in the bacterial solution of *A. rhizogenes* (K599, MSU440, and C58C1) and subjected to vacuum treatment using a laboratory vacuum desiccator for 30 min. For the second method, the bacterial solution was injected into the plant base using a disposable sterile syringe, ensuring that distinct droplets form at the injection site. For the third method, the root of the passion fruit seedling was cut off, and fresh *A. rhizogenes* strains harboring the recombinant plasmid were scraped from solid LB plates using a sterilized applicator and applied to the wound site. After inoculation, the seedlings were transplanted into a substrate (vermiculite:perlite:soil, 1:1:3 *v*/*v*/*v*), with the inoculated area covered by the substrate. The seedlings were covered with a transparent plastic dome and co-cultivated in the dark for 3 days. Following the co-cultivation period, the seedlings were transferred to normal light conditions and regularly watered to maintain moisture.

Transgenic hairy roots verification took place after 30 days. The number of plants which formed hairy roots were counted, and the transformation rate was calculated. The transgenic hairy roots were identified using a GFP fluorescent flashlight (LUYOR-3415RG), with an excitation wavelength of 440nm and an emission wavelength of 500 nm. The positive rate of hairy root plants was calculated as follows: (Number of positive hairy root plants/Total number of treated plants) × 100. The GFP-positive roots were further confirmed using regular PCR amplification and the primers are listed in Table S1.

### 4.5. Determination of PA Content

The PA content was determined in accordance with the protocol of a plant Oligomeric Proantho Cyanidins kit (Suzhou Comin Biotechnology Co., Ltd., Suzhou, China). Briefly, approximately 0.1 g of samples were added to 2.0 mL of pre-chilled extraction buffer (60% ethanol) and the tissue was mechanically disrupted using a homogenizer until complete lysis was achieved. Then, the homogenate was incubated in a constant-temperature shaker at 60 °C for 2 h to facilitate the extraction. After centrifugation at 10,000× *g* for 10 min, the supernatant was collected for subsequent spectrophotometric analysis with the wavelength set as 500 nm. For the reaction system, 200 μL of the test supernatant and 800 μL of the working reagent (Reagent I (8% hydrochloric acid in methanol) mixed with Reagent II (vanillin in methanol) at a 1:1 ratio was set as the assay tube, while 200 μL of the test supernatant and 800 μL of blank working solution (Reagent I mixed with methanol at a 1:1 ratio) was set as the control tube. The ΔA was calculated as the absorbance of the assay tube minus that of the control tube. Based on the standard curve provided by the kit (y = 0.0194x + 0.0006, R^2^ = 0.999), the proanthocyanidin content was converted using the following formula: Proanthocyanidin content (mg/g fresh weight) = 0.515× (ΔA − 0.0006) ÷ fresh weight of sample (g).

### 4.6. Direct Observation of Transgenic Hairy Roots

The red visual marker vector RUBY plasmids were transformed into *A. rhizogenes* strain K599 by the freeze–thaw method. Then, the *A. rhizogenes* was introduced into passion fruit using the optimized system, with OD_600_ = 0.6, infection duration 30 min, acetosyringone concentration 100 μM, and a dark co-cultivation period of 2 days. After one month, the transgenic hairy roots were identified by observing the emergence of red hairy roots.

### 4.7. Statistical Analysis

Each experiment was conducted at least three times independently. Statistical significance was determined by analysis of an ANOVA (Turkey’s test) using IBM SPSS Statistics 21 software. Values followed by different letters were significantly different at *p* < 0.05 and asterisks (**) were significantly different at *p* < 0.01.

## 5. Conclusions

Here, we successfully developed a rapid and efficient root transgenic system for passion fruit. The optimal parameters were determined as follows: infection with *A. rhizogenes* strain K599 at an OD_600_ of 0.6 for 30 min, with an AS concentration of 100 μM, followed by co-cultivation for 2 days. To validate the utility of this transformation system for studying gene function in transgenic hairy roots, we generated transgenic roots overexpressing *PeMYB123*, a key transcription factor known to regulate proanthocyanidin biosynthesis in passion fruit. The transgenic roots exhibited a significant increase in proanthocyanidin content, accompanied by upregulation of the proanthocyanidin-related gene *PeANR*. These findings provide a simple and efficient method for gene function analysis in passion fruit, offering valuable insights into the molecular mechanisms underlying secondary metabolism in this important crop.

## Data Availability

Data are contained within the article and Supplementary Materials.

## Supplementary Materials

The following supporting information can be downloaded at: https://www.mdpi.com/article/10.3390/plants14152312/s1. Figure S1. The morphology of hairy roots; Figure S2: Phylogenetic analysis of PeMYB123; Figure S3: Phylogenetic tree derived from amino acid sequences of PeMYB123 and other MYBs involved in flavonoid biosynthesis; Table S1. The primers used in this study.

## References

[1] Faleiro F.G., Junqueira N.T.V., Junghans T.G., Jesus O.N.D., Miranda D., Otoni W.C. (2019). Advances in passion fruit (*Passiflora* spp.) propagation. Rev. Bras. Frutic..

[2] Pereira Z.C., Cruz J.M.D.A., Correa R.F., Sanches E.A., Campelo P.H., Bezerra J.A. (2023). Passion fruit (*Passiflora* spp.) pulp, A review on bioactive properties, health benefits and technological potential. Food Res. Int..

[3] Shad M.A., Wu S., Rao M.J., Luo X., Huang X., Wu Y., Zhou Y., Wang L., Ma C., Hu L. (2024). Evolution and functional dynamics of TCP transcription factor gene family in passion fruit (*Passiflora edulis*). Plants.

[4] Fonseca A.M.A., Geraldi M.V., Junior M.R.M., Silvestre A.J.D., Rocha S.M. (2022). Purple passion fruit (*Passiflora edulis* f. *edulis*), A comprehensive review on the nutritional value, phytochemical profile and associated health effects. Food Res. Int..

[5] Gadioli I.L., Correa R.C.G., Barros L., Calhelha R.C., Alves M.J., Abreu R.M.V., Santos-Buelga C., Ferreira I.C.F.R. (2018). A systematic review on phenolic compounds in *Passiflora* plants, exploring biodiversity for food, nutrition, and popular medicine. Crit. Rev. Food Sci. Nutr..

[6] Rudnicki M., Silveira M.M., Pereira T.V., Oliveira M.R., Moreira J.C.F. (2007). Protective effects of *Passiflora alata* extract pretreatment on carbon tetrachloride induced oxidative damage in rats. Food Chem. Toxicol..

[7] Zhao Y., Jiang C., Lu J., Sun Y., Cui Y. (2023). Research progress of proanthocyanidins and anthocyanidins. Phytother. Res..

[8] Rauf A., Imran M., Abu-Izneid T., Iahtisham-Ul-Haq I., Patel S., Pan X., Naz S., Sanches Silva A., Saeed F., Rasul Suleria H.A. (2019). Proanthocyanidins, A comprehensive review. Biomed. Pharmacother..

[9] Domínguez-Rodríguez G., Plaza M., Marina M.L. (2021). High-performance thin-layer chromatography and direct analysis in real time-high resolution mass spectrometry of non-extractable polyphenols from tropical fruit peels. Food Res. Int..

[10] Pugliese A.G., Tomas-Barberan F.A., Truchado P., Genovese M.I. (2013). Flavonoids, proanthocyanidins, vitamin C, and antioxidant activity of *Theobroma grandiflorum* (Cupuassu) pulp and seeds. J. Agric. Food Chem..

[11] Zeng Y.X., Wang S., Wei L., Cui Y.Y., Chen Y.H. (2020). Proanthocyanidins, Components, Pharmacokinetics and Biomedical Properties. Am. J. Chin. Med..

[12] Ferrer J.L., Austin M.B., Stewart C., Noel J.P. (2008). Structure and function of enzymes involved in the biosynthesis of phenylpropanoids. Plant Physiol. Biochem..

[13] Jiang W., Yin Q., Liu J., Su X., Han X., Li Q., Zhang J., Pang Y. (2024). The APETALA2-MYBL2 module represses proanthocyanidin biosynthesis by affecting formation of the MBW complex in seeds of Arabidopsis thaliana. Plant Commun..

[14] An X.H., Tian Y., Chen K.Q., Liu X.J., Liu D.D., Xie X.B., Cheng C.G., Cong P.H., Hao Y.J. (2015). MdMYB9 and MdMYB11 are involved in the regulation of the JA-induced biosynthesis of anthocyanin and proanthocyanidin in apples. Plant Cell Physiol..

[15] Zhang X., Ma W., Guan X., Wang F., Fan Z., Gao S., Yao Y. (2023). VvMYB14 participates in melatonin-induced proanthocyanidin biosynthesis by upregulating expression of VvMYBPA1 and VvMYBPA2 in grape seeds. Hortic. Res..

[16] Zhou H., Lin-Wang K., Liao L., Gu C., Lu Z., Allan A.C., Han Y. (2015). Peach MYB7 activates transcription of the proanthocyanidin pathway gene encoding leucoanthocyanidin reductase, but not anthocyanidin reductase. Front. Plant Sci..

[17] Su W., Xu M., Radani Y., Yang L. (2023). Technological Development and Application of Plant Genetic Transformation. Int. J. Mol. Sci..

[18] Wang P., Si H., Li C., Xu Z., Guo H., Jin S., Cheng H. (2025). Plant genetic transformation, achievements, current status and future prospects. Plant Biotechnol. J..

[19] Rahman S.U., Khan M.O., Ullah R., Ahmad F., Raza G. (2024). Agrobacterium-Mediated Transformation for the Development of Transgenic Crops; Present and Future Prospects. Mol. Biotechnol..

[20] Tiwari M., Mishra A.K., Chakrabarty D. (2022). Agrobacterium-mediated gene transfer, recent advancements and layered immunity in plants. Planta.

[21] Schröpfer S., Lempe J., Emeriewen O.F., Flachowsky H. (2022). Recent Developments and Strategies for the Application of Agrobacterium-Mediated Transformation of Apple *Malus* × *domestica* Borkh. Front. Plant Sci..

[22] Conti G., Xoconostle-Cázares B., Marcelino-Pérez G., Hopp H.E., Reyes C.A. (2021). Citrus Genetic Transformation, An Overview of the Current Strategies and Insights on the New Emerging Technologies. Front. Plant Sci..

[23] Ren C., Lin Y., Liang Z. (2022). CRISPR/Cas genome editing in grapevine, recent advances, challenges and future prospects. Fruit. Res..

[24] Manders G., Otoni W.C., Vaz F.B.D., Blackhall N.W., Power J.B., Davey M.R. (1994). Transformation of passionfruit (*Passiflora edulis* fv *flavicarpa* Degener.) using Agrobacterium tumefaciens. Plant Cell Rep..

[25] Huang D., Wu B., Chen G., Xing W., Xu Y., Ma F., Li H., Hu W., Huang H., Yang L. (2024). Genome-wide analysis of the passion fruit invertase gene family reveals involvement of PeCWINV5 in hexose accumulation. BMC Plant Biol..

[26] Monteiro-Hara A.C., Jadao A.S., Mendes B.M.J., Rezende J.A., Trevisan F., Mello A.P.O., Vieira M.L.C., Meletti L.M.M., De Piedade S.M. (2011). Genetic transformation of passionflower and evaluation of R1 and R2 generations for resistance to Cowpea aphid borne mosaic virus. Plant Dis..

[27] Trevisan F., Mendes B.M.J., Maciel S.C., Vieira M.L.C., Meletti L., Rezende J.A.M. (2006). Resistance to Passion fruit woodiness virus in transgenic passionflower expressing the virus coat protein gene. Plant Dis..

[28] Loyola-Vargas V.M., Méndez-Hernández H.A., Quintana-Escobar A.O. (2024). The History of Agrobacterium Rhizogenes, From Pathogen to a Multitasking Platform for Biotechnology. Methods Mol. Biol..

[29] Shi M., Liao P., Nile S.H., Georgiev M.I., Kai G. (2021). Biotechnological Exploration of Transformed Root Culture for Value-Added Products. Trends Biotechnol..

[30] Gantait S., Mukherjee E. (2021). Hairy root culture technology, applications, constraints and prospect. Appl. Microbiol. Biotechnol..

[31] Liu L., Qu J., Wang C., Liu M., Zhang C., Zhang X., Guo C., Wu C., Yang G., Huang J. (2024). An efficient genetic transformation system mediated by *Rhizobium rhizogenes* in fruit trees based on the transgenic hairy root to shoot conversion. Plant Biotechnol. J..

[32] Yan H., Ma D., Yi P., Sun G., Chen X., Yi Y., Huang X. (2023). Highly efficient *Agrobacterium rhizogenes*-mediated transformation for functional analysis in woodland strawberry. Plant Methods.

[33] Qin Y., Wang D., Fu J., Zhang Z., Qin Y., Hu G., Zhao J. (2021). *Agrobacterium rhizogenes*-mediated hairy root transformation as an efficient system for gene function analysis in Litchi chinensis. Plant Methods.

[34] Fang T., Zheng Y., Ma Q., Ren R., Xian H., Zeng L. (2025). Integrated Transcriptomic and Metabolomic Analysis Revealed Regulatory Mechanisms on Flavonoids Biosynthesis in the Skin of Passion Fruit (*Passiflora* spp.). J. Agric. Food Chem..

[35] Grimplet J. (2022). Genomic and Bioinformatic Resources for Perennial Fruit Species. Curr. Genom..

[36] Fang T., Wang M., He R., Chen Q., He D., Chen X., Li Y., Ren R., Yu W., Zeng L. (2024). A 224-bp Indel in the Promoter of *PeMYB114* Accounts for Anthocyanin Accumulation of Skin in Passion Fruit (*Passiflora* spp.). J. Agric. Food Chem..

[37] Ren R., Chen Y., Yu X., Peng X., Zeng L., Fang T. (2025). Identification and characterization of SWEET gene family in passion fruit reveals the involvement of *PeSWEET3* in soluble sugar accumulation. Plant Physiol. Biochem..

[38] Yin M., Jiang Y.H., Wen Y.J., Shi F.C., Huang H., Yan Q., Liu H.L. (2025). Establishment of an efficient *Agrobacterium rhizogenes*-mediated hairy root transformation method for subtropical fruit trees. Hortic. Plant J..

[39] Hou Y., Wong D.C.J., Sun X., Li Q., Zhou H., Meng L., Liao X., Liang Z., Aryal R., Wang Q. (2024). VvbHLH036, a basic helix-loop-helix transcription factor regulates the cold tolerance of grapevine. Plant Physiol..

[40] Zhang S.Y., Luo R.F., Wu Y.X., Zhang T.T., Yusuf A., Wang N., Li M., Duan S. (2025). Establishment and application of high-pressure propagation breeding (HPPB)-mediated genetic transformation system in citrus rootstocks. Plant Biotechnol. J..

[41] Otoni W.C., Soares J.R., Souza C.S., Silva L.A.S., Dias L.L.L., Robledo K.J.M., Paim-Pinto D.L., Koehler A.D., Sodrzeieski P.A., Fernandes A.M. (2024). Advances in Tissue Culture and Transformation Studies in Non-model Species, *Passiflora* spp. (Passifloraceae). Methods Mol. Biol..

[42] Rizwan H.M., Yang Q., Yousef A.F., Zhang X., Sharif Y., Kaijie J., Shi M., Li H., Munir N., Yang X. (2021). Establishment of a Novel and Efficient *Agrobacterium*-Mediated in Planta Transformation System for Passion Fruit (*Passiflora edulis*). Plants.

[43] Ajdanian L., Niazian M., Torkamaneh D. (2024). Optimizing ex vitro one-step RUBY-equipped hairy root transformation in drug- and hemp-type Cannabis. Plant Biotechnol. J..

[44] Zhou H., Hou Y., Tan L., Li Q., Li W., Kafle S., Xu M., Kiselev K.V., Meng L., Xin H. (2025). VaEIN3.1-VaERF057-VaFBA1 Module Positively Regulates Cold Tolerance by Accumulating Soluble Sugar in Grapevine. Plant Cell Environ..

[45] Bahramnejad B., Naji M., Bose R., Jha S. (2019). A critical review on use of *Agrobacterium rhizogenes* and their associated binary vectors for plant transformation. Biotechnol. Adv..

[46] Meng D., Yang Q., Dong B., Song Z., Niu L., Wang L., Cao H., Li H., Fu Y. (2019). Development of an efficient root transgenic system for pigeon pea and its application to other important economically plants. Plant Biotechnol. J..

[47] Xie X.T., Huang Q.Y., Wen G.C., Yuan H.W., He Y., Yan D.L., Huang J.Q., Wang X.F., Zheng B.S. (2022). Construction of *Agrobacterium rhizogenes*-mediated transformation system of *Carya illinoinensis* without dependence on tissue culture. J. Fruit Sci..

[48] Bhagat P., Verma S.K., Singh A.K., Aseri G.K., Khare N. (2019). Evaluation of influence of different strains of *Agrobacterium rhizogenes* on efficiency of hairy root induction in Rauwolfia serpentina. Indian J. Genet. Pl. Br..

[49] Cheng Y., Wang X., Li C., Ji J., Liu T., Duan K. (2021). Highly efficient *Agrobacterium rhizogenes*-mediated hairy root transformation for gene functional and gene editing analysis in soybean. Plant Methods.

[50] Yu W.L., Yang L., Xiang Y.Y., Li R.D., Zhou X.Q., Gan L.C., Xiang X.Y., Zhang Y.Y., Yuan L., Luo Y.Q. (2024). Development of a rapid and efficient system for CR genes identification based on hairy root transformation in Brassicaceae. Hortic. Plant J..

[51] Kumar V., Sharma A., Prasad B., Gururaj H., Ravishankar G. (2006). *Agrobacterium rhizogenes* mediated genetic transformation resulting in hairy root formation is enhanced by ultrasonication and acetosyringone treatment. Electron. J. Biotechnol..

[52] Wang Y., Wang J., Luo D., Jia J. (2001). Regeneration of plants from callus cultures of roots induced by *Agrobacterium rhizogenes* on *Alhagi pseudoalhagi*. Cell Res..

[53] Niazian M., Belzile F., Curtin S., de Ronne M., Torkamaneh D. (2023). Optimization of in vitro and ex vitro *Agrobacterium rhizogenes*-mediated hairy root transformation of soybean for visual screening of transformants using RUBY. Front. Plant Sci..

[54] Yi X., Sun X., Tian R., Li K., Ni M., Zhang Z., Liu J., Wang Q., Ma Q., Li J. (2022). Genome-wide characterization of the aquaporin gene family in radish and functional analysis of RsPIP2-6 involved in salt stress. Front. Plant Sci..

[55] Plasencia A., Soler M., Dupas A., Ladouce N., Silva-Martins G., Meilan R., Marron N., Jouanin L. (2016). Eucalyptus hairy roots, a fast, efficient and versatile tool to explore function and expression of genes involved in wood formation. Plant Biotechnol. J..

[56] Kai G., Xu H., Zhou C., Liao P., Xiao J., Luo X., You L., Zhang L. (2011). Metabolic engineering tanshinone biosynthetic pathway in *Salvia miltiorrhiza* hairy root cultures. Metab. Eng..

[57] Hao X., Pu Z., Cao G., You D., Zhou Y., Deng C., Shi M., Nile S.H., Wang Y., Zhou W. (2020). Tanshinone and salvianolic acid biosynthesis are regulated by SmMYB98 in *Salvia miltiorrhiza* hairy roots. J. Adv. Res..

[58] Ma H., Liu N., Sun X., Zhu M., Mao T., Huang S., Meng X., Li H., Wang M., Liang H. (2023). Establishment of an efficient transformation system and its application in regulatory mechanism analysis of biological macromolecules in tea plants. Int. J. Biol. Macromol..

[59] Ramasamy M., Dominguez M.M., Irigoyen S., Padilla C.S., Mandadi K.K. (2023). *Rhizobium rhizogenes*-mediated hairy root induction and plant regeneration for bioengineering citrus. Plant Biotechnol. J..

