# Supplementary material for: Establishment of an Efficient Agrobacterium rhizogenes-Mediated Hairy Root Transformation System for Functional Analysis in Passion Fruit
Source: Plants (Basel). 2025 Jul 26;14(15):2312. doi: 10.3390/plants14152312 (PMC12349569; doi:10.3390/plants14152312)
Supplement: Supplementary file 1 [file plants-14-02312-s001.zip › Supplemental Figure.pdf]

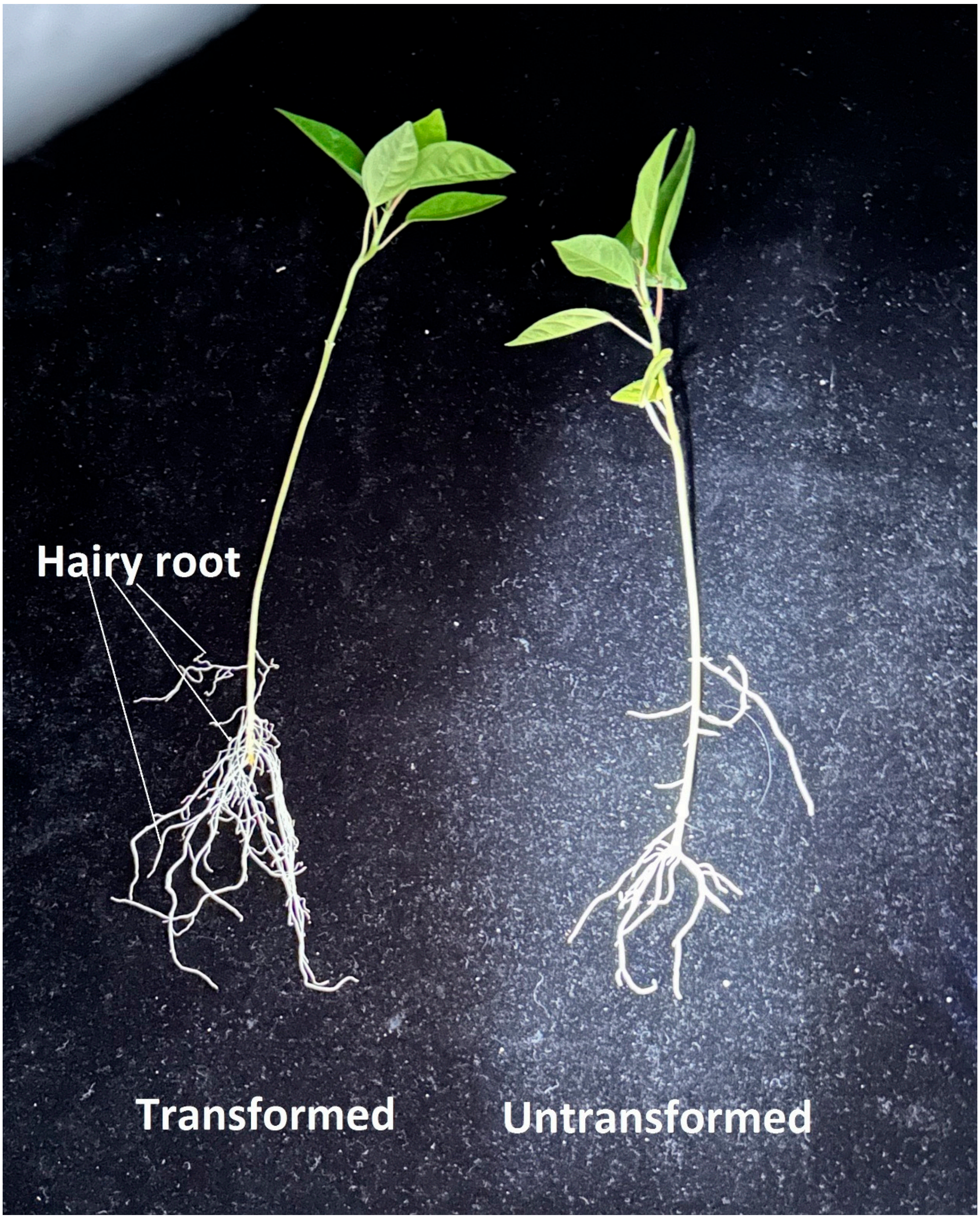

Figure S1. The morphology of hairy roots.

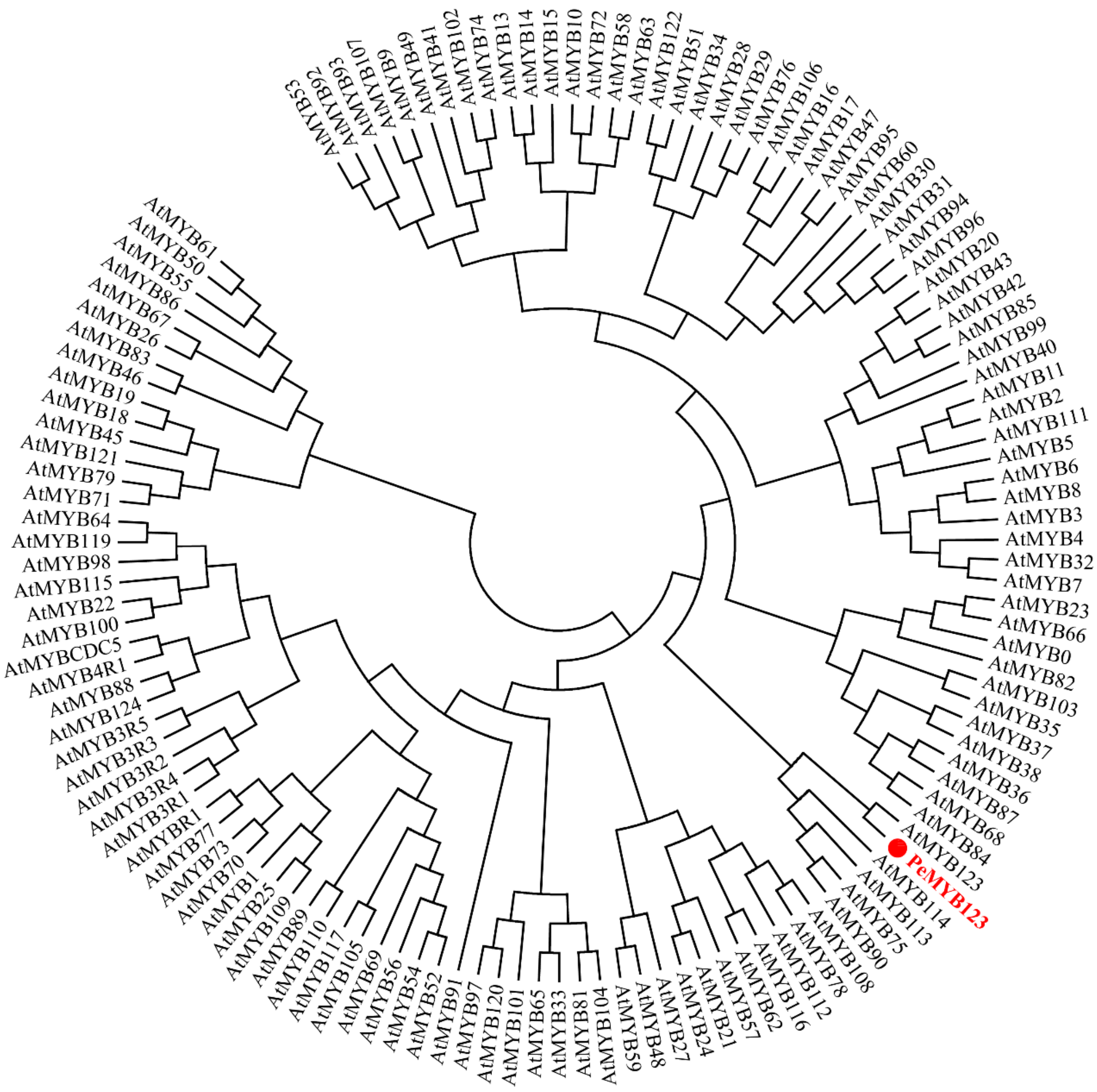

Figure S2. Phylogenetic analysis of PeMYB123.

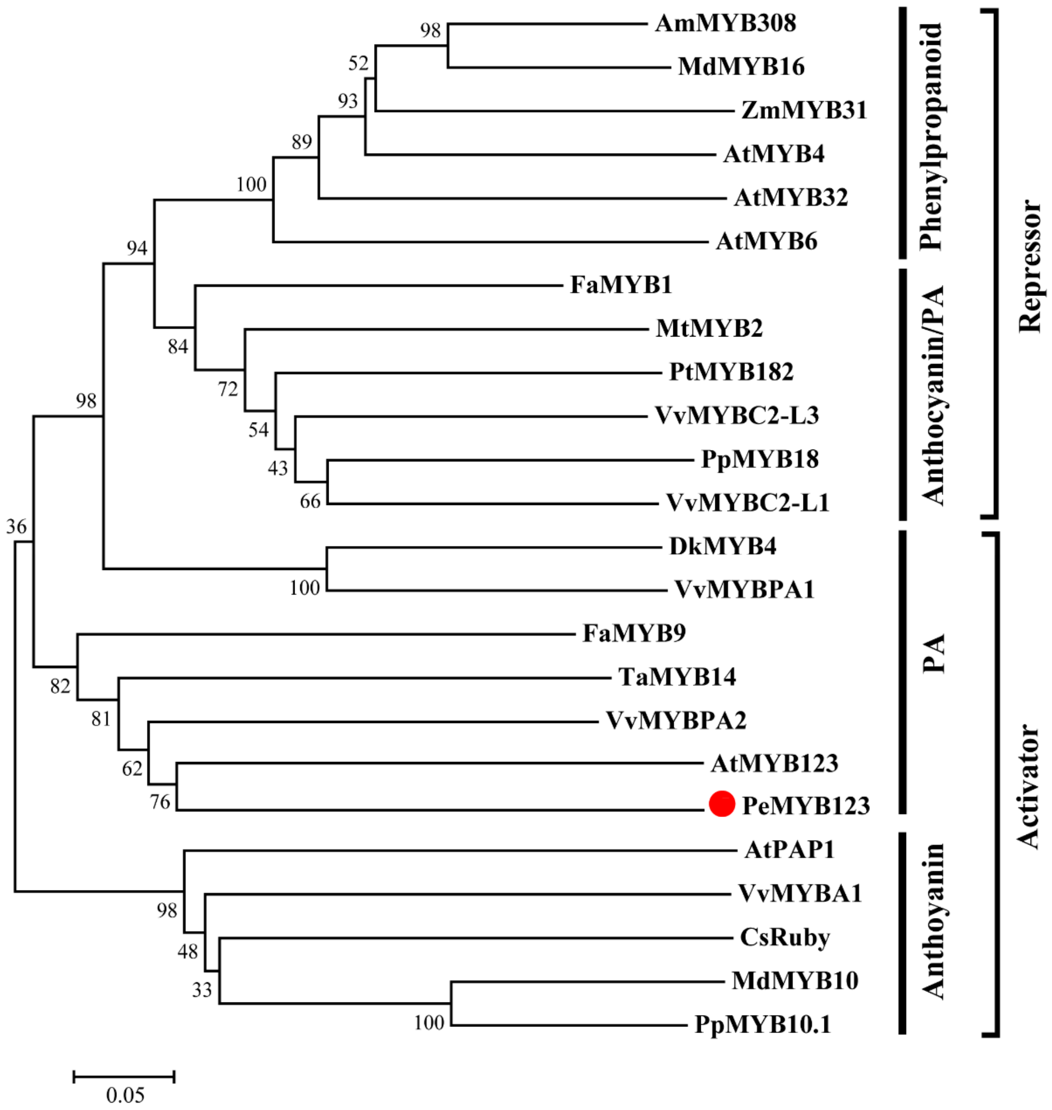

Figure S3. Phylogenetic tree derived from amino acid sequences of PeMYB123 and other MYBs involved in flavonoid biosynthesis.

PeMYB123 was highlighted with the solid red circle. Numbers indicate bootstrap test result with 1000 replicate analyses. The scale bar represents 0.5 substitutions per site. The following GenBank or Arabidopsis TAIR accession numbers were used: *Arabidopsis thaliana* AtMYB4 (AT4G38620), AtMYB6 (AT4G09460), AtPAP1 (AT1G56650), AtMYB123 (AT5G35550), AtMYB32 (AT4G34990); *Vitis vinifera* VvMYBA1 (AB097923), VvMYBPA2 (EU919682), VvMYBPA1 (AM259485), VvMYBC2-L1 (JX050227), VvMYBC2-L3 (KM046932); *Prunus persica* PpMYB18 (KT159234), PpMYB10.1 (XM_007216468); *Malus domestica* MdMYB10 (DQ267897), MdMYB16 (HM122617); *Fagaria ananassa* FaMYB9 (JQ989281), FaMYB1 (AF401220); *Diospyros kaki* DkMYB4 (AB503701); *Trifolium arvense* TaMYB14 (JN049641); *Citrus sinensis* CsRuby (NM_001288889); *Antirrhinum majus* AmMYB308 (P81393); *Populus tremula* PtMYB182 (KP723392); *Medicago truncatula* MtMYB2 (XM_003616340) and *Zea mays* ZmMYB31 (NP_001105949).
